# A rare case of juvenile hypertension: coexistence of type 2 multiple endocrine neoplasia -related bilateral pheochromocytoma and reninoma in a young patient with ACE gene polymorphism

**DOI:** 10.1186/s12902-015-0022-5

**Published:** 2015-06-18

**Authors:** Rosa Maria Paragliola, Ettore Capoluongo, Francesco Torino, Angelo Minucci, Giulia Canu, Alessandro Prete, Alfredo Pontecorvi, Salvatore Maria Corsello

**Affiliations:** Endocrinology Unit, Università Cattolica del Sacro Cuore, Largo Agostino Gemelli 8, 00168 Rome, Italy; Biochemistry and Clinical Biochemistry Unit, Università Cattolica del Sacro Cuore, Rome, Italy; Department of Systems Medicine, Università degli Studi Tor Vergata, Rome, Italy

**Keywords:** Pheochromocytoma, Reninoma, MEN2B, ACE polymorphism, Hypertension

## Abstract

**Background:**

Pheochromocytoma and reninoma represent two rare diseases causing hypertension. We here reported a rare case of association between type 2 multiple endocrine neoplasia related bilateral pheochromocytoma and reninoma. Moreover, polymorphism of ACE gene, which is known to be related to an increase of cardiovascular risk, has been found in the same patient.

**Case presentation:**

A 24 year old Caucasian man came to our attention for severe hypertension, resistant to anti-hypertensive polytherapy. At the age of twenty he had undergone total thyroidectomy with lymphadenectomy for medullary carcinoma. Genetic testing showed a *RET* mutation of codon 918 (exon 16) not documented in other family members. During the follow-up, a progressive increase of urinary metanephrines and catecholamines was recorded. Our evaluation confirmed the presence of severe hypertension (220/140 mmHg) and a severe increase of urinary catecholamines and metanephrines. Due to the presence of hypokalemia, other causes of hypertension were researched leading to the discovery of hyperreninemia (236 μUI/ml) with mild hyperaldosteronism, and a mild increase of the renal artery resistance at ultrasound. An abdominal MRI showed multiple adrenal masses and a right kidney nodular lesion of about 2 cm.

The patient underwent bilateral adrenalectomy and right nephrectomy, and histology confirmed the presence of bilateral pheochromocytoma and right reninoma. The post-surgery laboratory evaluation showed a rapid reduction of the urinary metanephrines while plasma renin level remained low in spite of the bilateral adrenalectomy without any mineralocorticoid supplementation. To further investigate these unusual feature, we performed genetic testing for the ACE gene, which revealed the presence of ACE I/D polymorphism.

**Conclusion:**

This unique report describes the association between two rare causes of hypertension in the same patient. Furthermore, the absence of requirement of mineralocorticoid supplementation in spite of bilateral adrenalectomy, represent an uncommon and interest finding.

## Background

Pheochromocytoma (PHEO) and reninoma both represent two rare causes of juvenile hypertension. PHEO is a neural crest-derived tumor, which produces and secretes catecholamines and which is related to the classical clinical symptoms associated with the inappropriate catecholamines secretion, such as are paroxysmal hypertension, tachycardia, palpitations, headaches and sweating [1]. Even if PHEO is a rare disease (less than 0.2% of patients with hypertension [2]) the related cardiovascular symptoms have a significant morbidity and even mortality. PHEO can occur sporadically or as hereditary form related to genetic background. In particular, type 2 multiple endocrine neoplasia (MEN2) is an autosomal dominant inherited endocrine malignancy syndrome, due to germline mutations in the REarranged during Transfection (*RET*) proto-oncogene, which encodes a tyrosine kinase receptor involved in cell growth, differentiation and survival. MEN syndrome includes the following three subtypes: MEN2A (association between medullary thyroid carcinoma [MTC], PHEO and primary hyperparathyroidism); familial medullary thyroid cancer and MEN2B. The latter represents a less common but more aggressive form and it is characterized by the association between MTC and PHEO with other particular features such as mucosal neuromas, intestinal ganglioneuromas, and marfanoid habitus with skeletal deformations and joint laxity [3].

Reninoma is a rare juxtaglomerular cell tumor causing renin-mediated hypertension. This condition is usually diagnosed in adolescents and young adults and is typically characterized by sustained hypertension well responding to antihypertensive treatment targeting the renin-angiotensin-aldosterone (RAA) system [4].

We here described a unique case of association between bilateral MEN2B related PHEO in whom also a reninoma causing secondary hyperaldosteronism was diagnosed.

## Case presentation

### Case report

In March 2010 a 24 year old Caucasian man with marphanoid habitus (height: 175 cm; weight: 65 Kg) was referred to us for severe hypertension that was resistant to beta blockers, angiotensin converting enzyme (ACE) inhibitors, and diuretics. Four years earlier he had undergone total thyroidectomy with lymphadenectomy due to MTC (pT4N+; stage III UICC 2009). The preoperative diagnostic workup had excluded PHEO, and his family history was negative for MEN2B-related tumors. Genetic testing for germline mutations in *RET* proto-oncogene was not done. However, after his thyroid surgery a progressive increase of the urinary metanephrines and catecholamines was noted, with absence of abnormal findings at the magnetic resonance imaging (MRI) and metaiodobenzylguanidine (MIBG) scan.

At our clinical examination, a severe systo-diastolic hypertension (220/140 mmHg) was observed, and mucosal neuromas of the tongue were found. Laboratory findings included severe hypokaliemia (potassium 2.5 mEq/L), hyperreninemia and mild hyperaldosteronism. Urinary catecholamines were also increased, as well as urinary metanephrines and vanil-mandelic acid. Inappropriate cortisol secretion was ruled out on the basis of the presence of normal serum and urinary free cortisol and detectable adrenocorticotropic hormone (ACTH) levels (Table 1). An ultrasound of the kidneys revealed a mass of approximately 2 cm at the right kidney with an increased artery resistance (0.88; normal value < 0.7). Furthermore, an enlargement of both adrenal glands was detected.

**Table 1:** Laboratory evaluation performed preoperatively, post-operatively and after 3 years of follow-up

	Preoperative value	Postoperative value	Value after 3 years of follow-up	Normal range
Reninemia (clinostatism)	236 μUI/ml	10 μUI/ml	25 μUI/ml	2.8–40 μUI/ml
Aldosterone (clinostatism)	536 pmol/L	267 pmol/L	186 pmol/L	55–415 pmol/L
Epinephrine	145 nmol/d	65 nmol/d	43 nmol/d	11–120 nmol/d
Norepinephrine	708 nmol/d	265 nmol/d	218 nmol/d	71–507 nmol/d
Metanephrine	15,681 nmol/d	310 nmol/d	225 nmol/d	253–1,720 nmol/d
Normetanephrine	6,932 nmol/d	479 nmol/d	615 nmol/d	491–2,430 nmol/d
Vanil-mandelic acid	15 mg/d	2 mg/d	3.6 mg/d	<7 mg/d
ACTH	7 pmol/L	16 pmol/L	21 pmol/L	2.2–12.2 pmol/L
Serum cortisol	370 nmol/L	<14 nmol/L	<14 nmol/L	221–607 nmol/L
Urinary free cortisol	185 nmol/d	Not performed	Not performed	102–375 nmol/d

ACTH: adrenocorticotropic hormone; d: 24 h

The genetic test for *RET* germline mutations showed the substitution of methionine for threonine in exon 16 at codon 918 (p.M918T) which was not present in other family members (father, mother and sister). A recurrence of MTC was excluded through assessment of serum markers, showing undetectable levels of serum calcitonin and normal carcinoembryonic antigen (CEA) levels, and an ultrasound of the neck which was negative. Replacement therapy with levothyroxine (150 mcg/day) appeared to be adequate and post-surgical hypoparathyroidism was well controlled with calcitriol (0.5 mcg/day).

An abdominal MRI confirmed the presence of adrenal bilateral hyperplasia with multiple adrenal masses (16 and 18 mm in the right gland, and 10 and 15 mm in the left gland), and the right kidney nodular lesion of 20 x 20 mm, hyperintensive in T2-weighted sequences. MIBG scintigraphy showed bilateral adrenal uptake, strongly suggestive for bilateral PHEO. Thus, the patient started anti-hypertensive therapy with phenoxybenzamine 20 mg four-times daily, nifedipine slow release 30 mg twice-daily, and atenolol 50 mg once-daily. Blood pressure control eventually improved. An ecocardiography showed dilated cardiomyopathy and moderate systolic dysfunction (ejection fraction 46 %). One month later the patient underwent bilateral adrenalectomy and right nephrectomy. No hypertensive crisis occurred during the surgery. The tumors were soft brown-red well-circumscribed masses without cystic degeneration. Histology of the adrenal glands confirmed the presence of bilateral and multifocal adrenal PHEO. No vascular or capsular invasion was noted; mitotic figures were less than 3/10 high power field and no atypical mitoses were present. The Ki 67 index was 2 % and immunoistrochemistry was positive for chromogranin A and synaptophysin. The histology of the right kidney mass revealed a capsulated neoplasm with nests and diffused sheets of tumor cells interrupted by abundant vascularity in the form of capillary channels and sinusoids, which were suggestive for reninoma.

After surgery, glucocorticoid replacement therapy with hydrocortisone was immediately started together with adequate hydration. Anti-hypertensive therapy was withdrawn and blood pressure was low to normal. Laboratory evaluation showed a sudden reduction of urinary metanephrines and catecholamines. Serum ACTH levels increased accordingly to post-surgical hypoadrenalism while serum cortisol was undetectable, but renin levels were normal-low (10 μUI/ml), in the absence of mineralocorticoid supplementation. Currently, 3 years after surgery, the patient is tumor-free. During this follow-up, the patient has been treated with replacement therapy comprised of hydrocortisone 30 mg, levothyroxine 150 mcg, and calcitriol 0.5 mcg.

Because of the persistence of normal renin values and serum electrolytes (Table 1) and normal blood pressure values mineralocorticoid substitutive therapy was not proposed. Surprisingly, aldosterone serum levels were detectable in spite of bilateral adrenalectomy (Table 1). MRI evaluation performed during follow-up failed to reveal adrenal remnants which could justify these clinical and biochemical findings. Moreover, undetectable serum cortisol performed about 18 h after the last hydrocortisone administration, was extremely suggestive for the absence of adrenal remnants or for the presence of ectopic functioning adrenal tissue. To further investigate this unusual feature, we hypothesized that it could be due to a dysregulation of RAA axis. After obtaining the informed consent, we searched for ACE polymorphisms. Genetic test revealed ACE I/D polymorphism.

### Gene analysis

After obtaining informed consent, genomic DNA was isolated from peripheral blood by a manual method (Roche Diagnostics, Basel, Switzerland, http://www.roche.com/index.htm), while spectral analysis was performed spectrophotometrically in order to determine DNA concentration and purity (NanoPhotometer™, Implen, München Germany, http://www.implen.de). Mutation analysis of the *RET* gene was performed by sequencing of coding region and exon-intron boundaries of the exons 2, 5, 8, 10, 11, 13–16. Primers used and the respective annealing temperatures are reported in Table 2. PCR products were sequenced using the Big Dye Terminator v1.1 Cycle Sequencing kit (Applied Biosystems, Foster City, CA, USA, http://www.appliedbiosystems.com/absite/us/en/home.html) in an automated sequencer ABI Prism 3500 Genetic Analyzer (Applied Biosystems).

**Table 2:** Primers used for *RET* amplification and sequencing

Oligo Name	Sequence (5′ → 3′)	Annealing Temperature (Ta) [°C]
RET-2 F	GCT TCC CCT GTT TCC TTT TC	
RET-2R	AGT GTC AGC GGC TGT GAT AA	
RET-5 F	CGT GCA GCA TTC TAA GGT CTC	
RET-5R	CAT GTC TGT AGG GTG CTG CT	
RET-8 F	TGC TCC TGG CAC TGT CTT	
RET-8R	TGG GGA CCA ATC ACT GTA CTC	
RET-10 F	GGA CAC TGC CCT GGA AAT A	
RET-10R	ACT CGC CTC CCA GCA ATT T	
RET-11 F	ATA CGC AGC CTG TAC CCA CT	
RET-11R	AAA TGG GGG CAG AAC ACA	58
RET-13 F	CCA TCC TGA CCT GGT ATG GT	
RET-13R	AAA CAG GGC AGG AGC AGT AG	
RET-14 F	GTC CAC CCC CTT ACT CAT TG	
RET-14R	GTG GTG AGC CAT AGC ATG G	
RET-15 F	CCC CCG GCC CAG GTC TC	
RET-15R	GCT CCA CTA ATC TTC GGT ATC TTT	
RET-16 F	TCT CCT TTA CCC CTC CTT CC	
RET-16R	CAG TGA GGG GGT CAT TGC	

Finally, using the SeqScape® Software v2.5, sequences were aligned to the reference sequence NG_007489.1.

For detecting *ACE I/D* polymorphism we followed the molecular method reported in literature [5].

## Discussion

We describe the unusual association between two very rare conditions causing juvenile hypertension, bilateral PHEO and reninoma, in a patient affected by MEN2B and ACE-polymorphism. The coexistence of PHEO and hyperreninemia secondary to renal artery stenosis has been previously reported [6], while only a paper, which described a 18 years follow-up in a family affected by MEN 2 B, found the association between adrenal medullary hyperplasia and renin-secreting juxtaglomerular tumor [7]. Furthermore, we discovered in this patient the presence of ACE gene I/D polymorphism which is known to be associated with cardiovascular risk.

The estimated incidence of cathecolamine secreting tumors is 2–8 per million, with the peak age of occurrence in the third to fifth decade of life [8]. Recent population based studies suggest that up to 32 % of patients have a germline mutation in one of the known common susceptibility genes [9]. In particular, mutations of *VHL*, *NF1* and *RET* cause well characterized cancer susceptibility syndrome (von Hippel Lindau, Neurofibromatosis Type 1 and MEN2, respectively).

These patients usually have other clinical characteristics of the associated syndrome at time of presentation.

Moreover, mutations of mitochondrial succinate dehydrogenase (SDH) complex subunits genes (SDHA, SDHB, SDHC, and SDHD) and one complex cofactor (SDHAF2) have been reported in head and neck and abdominal paragangliomas. Recently, other genes (TMEM127, MAX, HIF2A, EGLN1, KIF1B, H-RAS) have been added to the group of susceptibility genes involved in the development of paragangliomas/PHEO [10].

In our case, the association between MTC and PHEO, which was diagnosed about 4 years after the diagnosis of MTC, in addition to specific clinical features, was strongly suggestive for MEN2B. Germline mutations in *RET* codon 918 (M918T) were found to be related to the classic MEN2B phenotype in 95 % of cases [11] but patients often lack a family history of MTC and harbor a *de novo* mutation [12]. MEN 2B is the most severe form of MEN 2: in normal conditions, activation of the RET receptor involves interaction with different ligands which triggers RET dimerization and determinates tyrosine kinase activation. The M918T mutation can both induce a conformational change in the kinase catalytic core leading to the activation of RET without dimerization enhancing the intrinsic kinase activity and alter the substrate specificity of RET [13].

Genetic test performed in our patient showed the presence of M918T mutation (exon 16) confirming the diagnosis of MEN 2B, while screening performed in other family members was negative. This mutation of *RET* protoncogene was described as strongly associated with the onset of PHEO [14].

In this patient both the presence of a syndrome predisposing to catecholamine-secreting tumors and the onset of a severe juvenile hypertension, led to a work-up for PHEO. Biochemical evaluation for the diagnosis of cathecolamine secreting tumors is based on the measurements of fractionated metanephrines and cathecolamines in urine or plasma, the latter not available in our Institution. Urinary fractionated metanephrines and cathecolamines show high sensitivity (97 and 86 % respectively) and good specificity (69 and 88 % respectively) in the diagnosis of cathecolamine secreting tumors, while vanil-mandelic acid provides superior specificity (95 %) but lower sensitivity (64 %) [15].

About tumor localization, either computed tomography (CT) or MRI are recommended and can identify more than 95 % of tumors. However, the higher specificity in detecting PHEO is guaranteed by nuclear medicine techniques, which provides functional imaging. ^123^I-MIBG is a guanethidine analogue resembling norepinephrine. For this reason, MIBG scintigraphy is very useful and extremely specific for the diagnostic localization of PHEO [16]. In our patient a bilateral tumor, which can occur in about one third of MEN2 related PHEO, was found. However, the patient was also evaluated for all other possible causes of secondary hypertension, thus discovering renin-dependent hyperaldosteronism. Eventually a reninoma, suspected on the basis of biochemical evaluation and radiological findings, was confirmed by post-operative histology. Furthermore, a very particular finding in this case is the occurrence of ACE gene polymorphism detected post-operatively, which may represent an additional cause of cardiovascular risk.

ACE induces the catalytic conversion of angiotensin I to angiotensin II which represents a crucial step in the regulation of the RAA axis in turn involved in the regulation of blood pressure and serum electrolytes. However, ACE can cleave other many substrates, including bradykinin and substance P [17], and is probably responsible of other effects. ACE is widely distributed throughout the body, including central nervous system, surface of vascular endothelium and epithelium of the renal proximal tubule [17, 18]. This raises the question about a possible local effect of ACE in these sites. In fact, the multiple activities of ACE are demonstrated by the phenotype exhibited by ACE knock-out mice, which have profound hypotension, hypoplastic renal medulla, but also some other abnormalities (such as infertility) which are not present in knock-out mice for other components of RAA system [19].

*ACE* polymorphism, first described in 1990, seems to be one of the major factors influencing ACE levels. In their report, Rigat et al. [20] reported a 287 base pair Alu repeat in 16^th^ intron of *ACE* (17q23), which was termed I (insert) allele. Conversely, the lack of the repeat is D (deletion) allele. It has been demonstrated that subjects with D/D genotype have the highest serum ACE levels (50 % more relative to I/I), heterozygous individuals (genotype I/D) have intermediate ACE levels (25 % more relative to I/I) while homozygous I/I have the lowest ACE levels [21]. The D/D genotype was found more frequently in patients with myocardial infarction than in control subjects [22], but other studies showed no correlation with coronary disease or stroke [23]. The I/D genotype, which has been detected in our patient, is described in association with several condition as diabetic peripheral neuropathy [24], risk of recurrent miscarriage [25] as well as hypertension and cardiovascular disease.

A very important point, in our opinion, is the association between *ACE* polymorphism, MEN2B-related PHEO and reninoma, which can all contribute, through different pathways, to severe juvenile hypertension.

To our knowledge, there are not data available in the Literature to affirm the possibility of a common genetic background which could justify this association. In particular, the possibility that reninoma could represent another possible disorder in MEN 2 B syndrome can only be supposed in consideration of the reported cases. However, these findings do not justify the screening of hyperaldosteronism in all patients affected by MEN 2 B, unless severe concomitant hypokaliemia is present, as in our case.

Moreover, this patient represents a unique model of ACE I/D polymorphism in post-surgical hypoadrenalism, condition in which aldosterone secreting tissue is “virtually” absent. The absence of adrenal remnants or ectopic functioning adrenal tissue is supported by undetectable serum cortisol values after adequate pharmacological wash-out. Furthermore, postoperative radiological imaging excluded the presence of adrenal remnants. As well known, in patients who have undergone bilateral adrenalectomy, an increase of ACTH and renin levels is expected, and subjects need both glucocorticoid and mineralocorticoid replacement. In our patient, in spite of the predictable increase of ACTH values, renin levels were unexpectedly normal, while serum aldosterone remained detectable. The low-normal renin levels that were seen immediately after the removal of the reninoma may have been due to the persistent suppression of the normal renin secretion by the reninoma, overriding the stimulation due to the reduction of aldosterone. Surprisingly, the renin levels remained low-normal long after the removal of both adrenal glands and the reninoma and the patient did not need mineralocorticoid replacement therapy and did not experience hypotension or hyperkaliemia, as expected in a condition of mineralocorticoid defect. We can try to explain this particular phenomenon by considering a possible extra-adrenal aldosterone production. In fact, the existence of extra-adrenal sites of aldosterone synthesis, such as heart or brain, has been clearly demonstrated [26] and an endogenous corticosteroid biosynthesis after bilateral adrenalectomy has been described [27], even if the contribution of these sources to the circulating hormone levels is not completely known. In fact, while mRNA levels of aldosterone synthase (CYP11B2) are about 100 to 10,000 times lower in the normal human heart than in the human adrenal gland [28], the aldosterone synthase expression has been found to be elevated in patient with heart failure. It is well known that both inappropriate cathecolamine [29] and aldosterone [28] secretion *per se* cause cardiac remodeling, similarly to what has been found in our patient who developed dilated cardiomyopathy and impaired systolic function. While we cannot exclude that the heart failure may have resulted in an increase in aldosterone synthase expression, on the other hand it is difficult to determine whether the presence of the ACE I/D polymorphism may have played a contributing role in this setting, except that it likely worsened the degree of hypertension and reduced the response to ACE-inhibitors which had been used for several years to treat the patient.

A possible hypothesis involving the role of ACE I/D polymorphism in explaining this particular biochemical finding, is an increase of ACE activity in extra-adrenal aldosterone biosynthesis. In fact, tissue expressing a “local angiotensin-aldosterone axis”, such as kidneys, heart and blood vessels, can generate angiotensin II independently from systemic renin-angiotensin-aldosterone axis [30].

We cannot exclude that ACE I/D polymorphism can enhance the synthesis of angiotensin II in “local angiotensin-aldosterone axis”.

## Conclusion

In conclusion, we describe a unique association of 3 independent causes of hypertension. The most interesting points (other than the association between two rare causes of hypertension such as reninoma and bilateral MEN2B-related PHEO) are the persistence of detectable aldosterone levels in spite of bilateral adrenalectomy and the detection of ACE I/D polymorphism. We cannot confirm whether these two findings are related or randomly associated. Although the data seem to suggest the potential for an additional source of ectopic adrenal aldosterone production that could justify the presence of normal renin values and detectable aldosterone levels, the exact role of the concomitant ACE I/D polymorphism and its impact on the clinical features of this case are not known.

## Consent

Written informed consent was obtained from the patient for publication of this Case report. A copy of the written consent is available for review by the Editor of this journal.

## Abbreviations

PHEO: Pheochromocytoma
MEN2: type 2 multiple endocrine neoplasia
RET: REarranged during Transfection
MTC: Medullary thyroid carcinoma
RAA: Renin-angiotensin-aldosterone
ACE: Angiotensin converting enzyme
MRI: Magnetic resonance imaging
MIBG: Metaiodobenzylguanidine
CEA: Carcinoembryonic antigen
ACTH: Adrenocorticotropic hormone

## References

[1] Plouin PF, Amar L, Lepoutre C (2010). Phaeochromocytomas and functional paragangliomas: clinical management. Best Pract Res Clin Endocrinol Metab.

[2] Pacak K, Linehan WM, Eisenhofer G, Walther MM, Goldstein DS (2001). Recent advances in genetics, diagnosis, localization, and treatment of pheochromocytoma. Ann Intern Med.

[3] Torino F, Paragliola RM, Barnabei A, Corsello SM (2010). Medullary thyroid cancer: a promising model for targeted therapy. Curr Mol Med.

[4] Trnka P, Orellana L, Walsh M, Pool L, Borzi P (2014). Reninoma: an uncommon cause of renin-mediated hypertension. Front Pediatr.

[5] Minucci A, Canu G, Concolino P, Guarino D, Boccia S, Ficarra S (2014). DNA from buccal swab is suitable for rapid genotyping of angiotensin-converting enzyme insertion/deletion (I/D) polymorphism. Clin Chim Acta.

[6] Kota SK, Kota SK, Meher LK, Tripathy PR, Sruti J, Modi KD (2012). Pheochromocytoma with renal artery stenosis: a case-based review of literature. J Cardiovasc Dis Res.

[7] Sizemore GW, Carney JA, Gharib H, Capen CC (1992). Multiple endocrine neoplasia type 2B: eighteen-year follow-up of a four-generation family. Henry Ford Hosp Med J.

[8] DeLellis RA, Lloyd RV, Heitz PU, Eng C (2004). World health organization classification of tumours. Pathology and genetics of tumours of endocrine organs.

[9] Mannelli M, Castellano M, Schiavi F, Filetti S, Giacchè M, Mori L (2009). Clinically guided genetic screening in a large cohort of italian patients with pheochromocytomas and/or functional or nonfunctional paragangliomas. J Clin Endocrinolol Metab.

[10] Martins R, Bugalho MJ (2014). Paragangliomas/pheochromocytomas: clinically oriented genetic testing. Int J Endocrinol.

[11] Machens A, Ukkat J, Brauckhoff M, Gimm O, Dralle H (2005). Advances in the management of hereditary medullary thyroid cancer. J Intern Med.

[12] Raue F, Frank-Raue K (2009). Genotype-phenotype relationship in multiple endocrine neoplasia type 2. Implications for clinical management. Hormones.

[13] Gujral TS, Singh VK, Jia Z, Mulligan LM (2006). Molecular mechanisms of RET receptor-mediated oncogenesis in multiple endocrine neoplasia 2B. Cancer Res.

[14] Eng C, Clayton D, Schuffenecker I, Lenoir G, Cote G, Gagel RF (1996). The relationship between specific RET proto-oncogene mutations and disease phenotype in multiple endocrine neoplasia type 2. International RET mutation consortium analysis. JAMA.

[15] Zelinka T, Eisenhofer G, Pacak K (2007). Pheochromocytoma as a catecholamine producing tumor: implications for clinical practice. Stress.

[16] Vaidyanathan G (2008). Meta-iodobenzylguanidine and analogues: chemistry and biology. Q J Nucl Med Mol Imaging.

[17] Erdos EG, Skidgel RA (1987). The angiotensin I-converting enzyme. Lab Invest.

[18] Alhenc-Gelas F, Baussant T, Hubert C, Soubrier F, Corvol P. The angiotensin converting enzyme in the kidney. J Hypertens Suppl. 1989; 7:S9–S13 (discussion S14). 10.1097/00004872-198909007-00003 2559174

[19] Esther CR, Howard TE, Marino EM, Goddard JM, Capecchi MR, Bernstein KE (1996). Mice lacking angiotensin-converting enzyme have low blood pressure, renal pathology, and reduced male fertility. Lab Invest.

[20] Rigat B, Hubert C, Alhenc-Gelas F, Cambien F, Corvol P, Soubrier F (1990). An insertion/deletion polymorphism in the angiotensin I-converting enzyme gene accounting for half the variance of serum enzyme levels. J Clin Invest.

[21] Danser AH, Deinum J, Osterop AP, Admiraal PJ, Schalekamp MA (1999). Angiotensin I to angiotensin II conversion in the human forearm and leg. Effect of the angiotensin converting enzyme gene insertion/deletion polymorphism. J Hypertens.

[22] Cambien F, Poirier O, Lecerf L, Evans A, Cambou JP, Arveiler D (1992). Deletion polymorphism in the gene for angiotensin-converting enzyme is a potent risk factor for myocardial infarction. Nature.

[23] Agerholm-Larsen B, Nordestgaard BG, Tybjaerg-Hansen A (2000). ACE gene polymorphism in cardiovascular disease: meta-analyses of small and large studies in whites. Arterioscler Thromb Vasc Biol.

[24] Inanir A, Basol N, Karakus N, Yigit S (2013). The importance of association between angiotensin-converting enzyme (ACE) I/D polymorphism and diabetic peripheral neuropathy. Gene.

[25] Wang Z, Wang P, Wang X, He X, Wang Z, Xu D (2013). Significant association between angiotensin-converting enzyme gene insertion/deletion polymorphism and risk of recurrent miscarriage: A systematic review and meta-analysis. Metabolism.

[26] MacKenzie SM, Connell JM, Davies E (2012). Non-adrenal synthesis of aldosterone: a reality check. Mol Cell Endocrinol.

[27] Freel EM, Bernhardt R, Ingram M, Wallace AM, Fraser R, Davies E (2007). Endogenous corticosteroid biosynthesis in subjects after bilateral adrenalectomy. Clin Endocrinol (Oxf).

[28] Essick EE, Sam F. Cardiac hypertrophy and fibrosis in the metabolic syndrome: a role for aldosterone and the mineralocorticoid receptor. Int J Hypertens. 2011;346985. 10.4061/2011/346985 PMC3124304 21747976

[29] Prejbisz A, Lenders JW, Eisenhofer G, Januszewicz A (2011). Cardiovascular manifestations of phaeochromocytoma. J Hypertens.

[30] MacKenzie SM, Fraser R, Connell JM, Davies E (2002). Local renin-angiotensin systems and their interactions with extra-adrenal corticosteroid production. J Renin Angiotensin Aldosterone Syst.

